# Mentors Perspectives on Advancing Global Health Research through Better Mentorship

**DOI:** 10.4269/ajtmh.26-0182

**Published:** 2026-08-11

**Authors:** Davidson H. Hamer, Elizabeth Fine, Yemane Berhane, Claudia Hawkins, Joseph Makhema, Jacqui Miot, Sailesh Mohan, Aneesa Moolla, Mosa Moshabela, Candida Moshiro, Sikhulile Moyo, Robert L. Murphy, Jonah Musa, Nisha Nadesan-Reddy, Thumbi Ndung’u, Folasade Ogunsola, Collins Ouma, Douglas J. Perkins, Christopher R. Sudfeld, Bruno Sunguya, Elvis K. Tiburu, Nicola Wearne, Alemayehu Worku, Wafaie W. Fawzi

**Affiliations:** ^1^ Department of Global Health, Boston University School of Public Health, Boston, Massachusetts, USA;; ^2^ Section of Infectious Diseases, Department of Medicine, Boston University Chobanian & Avedisian School of Medicine, Boston, Massachusetts, USA;; ^3^ Boston University Center on Emerging Infectious Diseases, Boston, Massachusetts, USA;; ^4^ Department of Global Health and Population, Harvard T.H. Chan School of Public Health, Boston, Massachusetts, USA;; ^5^ Department of Epidemiology and Biostatistics, Addis Continental Institute of Public Health, Addis Ababa, Ethiopia;; ^6^ Robert J. Havey Institute for Global Health, Feinberg School of Medicine, Northwestern University, Chicago, Illinois, USA;; ^7^ Department of Medicine, Feinberg School of Medicine, Northwestern University, Chicago, Illinois, USA;; ^8^ Botswana Harvard Health Partnership, Gaborone, Botswana;; ^9^ Health Economics and Epidemiology Research Office, Faculty of Health Sciences, Witwatersrand, Johannesburg, South Africa;; ^10^ Centre for Chronic Disease Control, New Delhi, India;; ^11^ The Independent Institute of Education (IIEMSA), South Africa;; ^12^ University of Cape Town, Cape Town, South Africa;; ^13^ Department of Epidemiology and Biostatistics, School of Public Health and Social Sciences, Muhimbili University of Health and Allied Sciences, Dar es Salaam, Tanzania;; ^14^ Department of Pathology, Division of Medical Virology, Stellenbosch University, Cape Town, South Africa;; ^15^ School of Health Systems and Public Health, University of Pretoria, Pretoria, South Africa;; ^16^ Department of Immunology and Infectious Diseases, Harvard T.H. Chan School of Public Health, Boston, Massachusetts, USA;; ^17^ School of Allied Health Professions, Faculty of Health Sciences, University of Botswana, Botswana;; ^18^ Department of Obstetrics and Gynecology, College of Health Sciences, University of Jos, Nigeria;; ^19^ Discipline of Public Health Medicine, University of KwaZulu-Natal, Durban, South Africa;; ^20^ Africa Health Research Institute, Durban, South Africa;; ^21^ HIV Pathogenesis Programme, The Doris Duke Medical Research Institute, University of KwaZulu-Natal, Durban, South Africa;; ^22^ Ragon Institute of Massachusetts General Hospital, Massachusetts Institute of Technology and Harvard University, Cambridge, Massachusetts, USA;; ^23^ Institute of Infection, Immunity and Transplantation, University College London, London, United Kingdom;; ^24^ University of Lagos, Lagos, Nigeria;; ^25^ Department of Biomedical Sciences and Technology, School of Public Health and Community Development, Maseno University, Kenya;; ^26^ Department of Internal Medicine, Center for Global Health, Northwestern University, Chicago, Illinois, USA;; ^27^ Department of Community Health, School of Public Health and Social Sciences, Muhimbili University of Health and Allied Sciences, Dar es Salaam, Tanzania;; ^28^ Department of Biomedical Engineering, University of Ghana, Legon, Ghana;; ^29^ Division of Nephrology and Hypertension, Faculty of Health Sciences, University of Cape Town, Cape Town, South Africa

## Abstract

Effective mentorship is essential for advancing global health research, particularly in low- and middle-income countries (LMICs). This review synthesizes expert perspectives from a highly experienced group of global health research mentors. We identify the characteristics and core competencies of effective global health mentors, emphasizing adaptability, cultural competence, and equitable collaboration. Special attention is paid to addressing barriers faced by underrepresented groups in academia, highlighting the role of mentorship in career advancement and enhancing workforce diversity. We describe a variety of mentoring models, e.g., one-to-one, peer, group, and virtual, and mentoring tools and frameworks, evaluating their relevance to diverse global health contexts. This review emphasizes their potential for customization in LMICs and provides guidance for structuring effective mentor–mentee communication. Recognizing that sustainable impact extends beyond individual relationships, we present strategies for developing institutional mentorship programs, drawing on lessons from Ethiopia and Kenya. Key recommendations include capacity-building for mentors and mentees, institutional ownership of mentoring activities, mentor–mentee matching, accessible training, and regular evaluation linked to recognition and career advancement. The transition from structured mentorship to professional independence merits special consideration, including mechanisms for fostering ongoing collegiality and long-term career support. Our findings underscore the importance of structured, intentional mentorship for building research capacity, driving academic productivity, and advancing global health equity. We advocate for sustained investment by individuals and institutions, recommending adaptable mentorship frameworks and supportive cultures to foster emerging global health leaders. By institutionalizing best practices, global health research mentorship can contribute decisively to stronger health systems worldwide.

## INTRODUCTION

Mentorship, traditionally a professional relationship between a senior and junior colleague or trainee, is an essential process for helping early-career researchers strengthen their knowledge and scientific approach, address career challenges, and develop professionally.^1^ In recent years, there has been an increased focus on mentoring in global health research to strengthen collaborative partnerships and improve the quality of scientific output.^2^^,^^3^ Global health mentorship involves guiding researchers who work across diverse geographic, cultural, and institutional settings, often addressing complex health challenges in low- and middle-income countries (LMICs) while emphasizing cross-cultural competence, equitable collaboration, capacity-building, and ethical engagement with communities. Effective mentorship in this context supports not only individual professional development but also sustainable research ecosystems and stronger health systems with a global impact.

Prior to 1998, mentoring was prevalent in academic medicine and promoted to support career growth, though little was written about this topic.^4^ In the last two decades, the field has evolved substantially and has been rigorously defined, studied, monitored, and evaluated. It is now taught in virtually all academic medical centers.^5^ However, mentoring remains both under-recognized and undervalued as a factor important for academic promotion in both high-income countries (HICs) and LMIC institutions. Strong mentoring programs provide tangible benefits to mentees, mentors, and affiliated institutions. Mentees report improved career planning, advancement, work satisfaction, and personal growth. Mentors gain new insights, perspectives, peer recognition, and satisfaction from influencing the next generation. Institutions benefit from higher levels of engagement, work satisfaction, recruitment, retention, and wellness.^6^^–^^8^

The Fogarty International Center at the National Institutes of Health has supported global health research training for the last two decades through its Global Health Research and Scholars Program. This program, renamed the Launching Future Leaders in Global Health Research Training Program (LAUNCH), was renewed for a new 5-year cycle in 2022.^9^ This program supports 1-year mentored global health research training fellowships at universities, and biomedical and health research institutions in LMICs. Major research topics include HIV, maternal–child health and nutrition (MCHN), noncommunicable diseases (NCDs), and mental health.

The HBNU consortium, with four US academic institutions (Harvard University, Boston University, Northwestern University, and the University of New Mexico) and 21 partners in Africa, Asia, and Latin America, has been funded by the LAUNCH program since 2017. Consortium leadership collaborated to review a broad range of global health research mentoring aspects, including core mentorship competencies,^10^ mentoring resources and tools, successful country mentorship programs, and strategies to build institutional mentoring capacity. This review synthesizes the experiences of HBNU mentors in global health research mentoring and is designed to enhance the capacity of individual mentors and equip institutions with the tools and skills needed to deliver effective mentorship.

## MENTOR CHARACTERISTICS AND QUALIFICATIONS

Mentoring is a dynamic process that evolves over time to benefit all involved. Mentors serve as role models and facilitators for professional networking, and they should encourage independent behavior, including guiding the mentee in goal-setting. Several basic characteristics facilitate these roles, beginning with experience and knowledge, as well as being available, supportive, trustworthy, enthusiastic, encouraging, empathetic, and active listeners.^11^^–^^15^ Mentors must make a conscious and deliberate commitment to demonstrate enthusiasm for their mentees’ career progression, while recognizing the mentee as an individual with a private life and respecting the mentee’s boundaries.^5^

Furthermore, several core competencies are required for effective mentoring. Identifying competencies for mentoring in a global health context is important to 1) define the expertise required for effective mentoring in LMICs; 2) advocate for resources; 3) develop and disseminate institutional guidelines; 4) identify support for research mentoring; and 5) address mentor training needs.^16^^–^^19^ Building on mentoring capacities developed by other scientists and educators, core competencies for global health researchers have been recently described.^20^ These eight key competencies reflect a progressive mentoring process, beginning with foundational communication, continuing through goal-setting and skill assessment, and culminating in professional development and independence (Box 1).

**Box 1:** Global health mentoring competencies^10^

1. Maintaining effective communication involves actively listening and showing genuine interest in understanding mentees’ issues. It requires encouraging open dialogue and attentiveness to nonverbal cues while ensuring regular availability for meetings. Effective communication also encompasses empathetic and culturally sensitive interactions, collaboration with fellow mentors, and the provision of constructive feedback on written and oral work. 2. Aligning expectations and goals requires mentors to demonstrate altruism and enthusiasm throughout the mentoring process. By maintaining self-awareness, mentors can better guide their mentees in realistically setting research goals and planning projects. This competency involves helping mentees develop effective strategies to attain their goals and providing support in implementing these strategies and plans, ensuring mentees are well-equipped for success. 3. Assessing skills and knowledge entails understanding the relevant subject matter, though this is not strictly necessary to be competent in all research areas. Mentors must identify key knowledge and skill gaps within the mentoring team and proactively address these needs by recruiting co-mentors or referring mentees to experts for additional guidance when necessary. 4. Addressing diversity requires mentors to embrace partnerships with those of differing backgrounds, including gender, sexual orientation, culture, nationality, religious background, etc. Additionally, mentors should be able to recognize their implicit and explicit biases and be willing to engage in continued learning or training to overcome them. 5. Fostering independence involves guiding mentees in setting and achieving their research career goals. Mentors assist in identifying potential funding opportunities, providing networking opportunities, and stepping back into a supporting role when needed. Additionally, mentors encourage successful mentees to take on leadership roles when appropriate, further promoting their independence and growth. 6. Promoting professional development involves supporting mentees in cultivating diverse communication skills, including public speaking and writing. Mentors guide mentees in learning the dynamics of teamwork, fostering a collaborative spirit. Additionally, they help mentees prioritize tasks, manage their time efficiently, and achieve a balance between work and personal life. 7. Promoting professional integrity and ethics by serving as a role model through personal and professional behaviors. This also includes guiding mentees in the responsible conduct of research, covering both approvals and implementation. Mentors should acknowledge mentees’ contributions and show a commitment to continuous learning and a genuine interest in helping others grow. 8. Building resilience to challenges includes equipping mentees with skills to manage administrative duties and imparting problem-solving skills, emphasizing patience and resilience in the face of setbacks. They assist mentees in identifying financial and nonfinancial resources necessary to achieve their research goals and help mentees develop solutions to navigate barriers such as internal politics and local bureaucracy.

## WORKFORCE DEVELOPMENT AND REPRESENTATION

Early-career researchers may encounter difficulties finding mentors who understand their experiences. Women, individuals with disabilities, and those from historically underrepresented backgrounds often face unique challenges in accessing academic and research opportunities.^21^ For example, studies have shown that in many LMIC settings, women remain underrepresented in health research and tend to publish less frequently than their male counterparts.^20^^,^^22^ Contributing factors may include greater caregiving responsibilities, unequal leadership opportunities, and challenges accessing publishing support. In many research settings, these differences may stem from access to resources, support systems, and institutional frameworks that address varied needs.^23^ Mentorship can help bridge these gaps by fostering comprehensive responsive environments that promote professional development. Deliberative efforts targeting contextual gender gaps can help minimize the low proportion of qualified and senior female researchers in LMIC academic and research institutions. Such support systems ensure that all individuals, regardless of background, have enhanced opportunities to succeed in research careers.

In global health research, local contexts, including institutional capacity and historical precedents, can shape the mentoring experience. Mentors may benefit from understanding how these environments differ and facilitating the implementation of approaches that are empathetic, flexible, and responsive to a range of needs. Training programs can strengthen mentorship by equipping mentors with tools to support a broad range of challenges and experiences. Ultimately, mentors and institutions share responsibility for cultivating academic environments that support professional growth through adaptable and effective mentoring pathways.

## MENTORING MODELS AND TOOLS

There are several different types of mentoring models, with some of the most popular and effective described in Table 1.^24^ In conventional or one-to-one mentoring, the mentor has more expertise and may seem significantly higher in academic rank than the mentee. This can hinder the formation of rapport. Peer mentoring, which usually consists of two or more people at the same level (e.g., doctoral students, post-docs, or junior faculty) mentoring each other, eliminates the power difference. By having a reliable peer mentor, mentees feel less isolated and more secure and accepted, which in turn boosts their self-esteem and helps them succeed in their studies and personal lives throughout college life. By reducing stress and preventing dropping out, peer mentoring may benefit university, doctoral, and postdoctoral students. Potential mentees may find peer mentoring easier to access because there are more peers than experienced mentors, with less time constraints for peer mentors. With peer mentoring, there is a greater chance for empathy, a sense of equality, and competence. Students can also develop communication, critical thinking, and collaboration skills that are highly sought-after by being a peer mentor. Finally, peer mentoring programs are considered less costly than hierarchical mentoring in relative terms.^25^^–^^27^

**Table 1:** Mentoring models

Mentoring Model	Description
One-to-one mentoring	More experienced person offers guidance, advice, and help to a less experienced person in their academic or professional development.
Group mentoring	One mentor provides guidance and advice to several mentees. Mentees typically have a common or similar goal.
Team mentoring	Multiple mentors provide guidance and advice to one mentee.
E-mentoring	Relationship guided completely or in part using electronic communication, such as E-mail, text, webinar, or social media. Technology is also used to assist and/or improve the mentoring relationships that happen face-to-face.
Reverse mentoring	Junior faculty member mentors a senior faculty member. Senior mentees might increase their skill on a particular type of new methodology or technology while the junior faculty member may develop leadership skills and organizational knowledge.
Peer mentoring	Relationship between persons who are at equal level of experience and rank in which one person is more knowledgeable than the other in a certain area and can lend a hand along with knowledge acquisition, advancement of academic proficiency, and skills transfer. Peer mentoring may be a one-to-one relationship or done in a group. Peer relationships offer a higher level of mutual benefit, with both individuals exchanging support.

Adapted from Faculty Mentoring Models and Effective Practices, Hanover Research.^24^

A variety of tools to enhance the productivity and success of mentor-mentee relationships are available. In the health care sciences, several different mentoring tools and resources exist to support areas, such as communication, professional development, and mentor–mentee relationships, and in some cases provide guidance for institutional roles in mentorship. Recently, some toolkits have included guidance on developing a mentor network to help students explore their mentoring needs and visualize their mentor network, including roles of specific mentors across various domains and plans for expanding their mentoring network to accommodate any existing unmet mentoring needs.^28^

Selecting the appropriate tools to use depends on the needs and goals of the mentoring relationship, learning styles, and preferences. A variety of online toolkits provide activities, checklists, and suggestions. Most mentoring tools and toolkits have been designed and used in HICs, with very few specifically designed for use in LMIC settings or mentoring in global health contexts (although our review included only toolkits published in English). These tools, therefore, do not account for additional considerations, such as differences in cultural, social, and economic backgrounds between mentors and mentees, and competing academic responsibilities and priorities.^29^ A recent scoping review of global health mentoring toolkits found that almost all the toolkits identified were developed in North America.^29^ Mentoring tools are being increasingly used and adapted into local contexts in LMICs as mentoring takes on greater significance, partly because of an increasing number of research training programs. Few existing tools provide guidance on how they can be adapted for use in these settings.^1^

Table 2 describes an “ideal set” of mentoring tools, which can be categorized by intended user. These include resources for mentees to help with goal-setting, identifying needs, assessing competencies, evaluation, aligning expectations, and effective communications.

**Table 2:** Mentoring tools and resources

Mentoring Tools and Resources	Examples of Tools and Resources	Resources or Toolkits	Description
Mentoring goals and objectives			
Development plan and goals	Individual development plan and timeline	https://cornell.app.box.com/v/ FAIM-tools-resources/file/1652450999007 https://ictr.wisc.edu/mentoring/individual-development-plan/ https://health.ucdavis.edu/media-resources/ctsc/documents/pdfs/idp-template-general.pdf	Tool designed to assist with: 1) Identifying professional goals and objectives 2) Assessing an individual’s skill set relative to their career goals 3) Developing a plan to acquire skills and competencies needed to achieve short- and long-term career objectives
Mentor–Mentee Relationship			
Self-assessment (mentee)	Self-assessment tool	https://medschool.duke.edu/sites/default/files/2021-12/mentee-self-assessment-worksheet1.pdf	Allows self-assessment of skills, abilities, strengths, and weaknesses as a mentee
Self-reflection (mentor)	Self-assessment tool	https://case.edu/provostscholars/sites/default/files/2019-09/OSU%20Mentoring-mentor-toolkit-%20final-508.pdf, p. 8 https://medicine.iu.edu/faculty/professional-development/mentoring/toolkit/mentors https://medschool.duke.edu/sites/default/files/2021-12/indiana_university_mentor_self_assessment_form.pdf https://uwmadison.qualtrics.com/jfe/form/SV_5jMT4fhemifK01n?Q_JFE=qdg (mentoring competency form)	Allows mentor to identify personal skills, abilities, strengths, and weaknesses with the aim of helping the mentor to understand what characteristics they possess that will allow them to be a good mentor
Mentee-focused needs assessment	Mentee tool	https://faculty.weill.cornell.edu/mentoring-tools https://cornell.app.box.com/v/FAIM-tools-resources	Helpful for mentee self-assessment
Mentor-focused needs assessment	Mentor tool	https://faculty.weill.cornell.edu/mentoring-tools	Designed to help mentors determine mentoring needs
Mentoring expectations of goals	Mentorship agreement forms Partnership compacts Mentor/mentee expectations scales worksheet	https://bwhmentoringtoolkit.partners.org/wp-content/uploads/2012/11/BWH-Mentoring-Agreement-12_14.pdf https://medicine.iu.edu/faculty/professional-development/mentoring/toolkit/documents https://medicine.iu.edu/faculty/professional-development/mentoring/toolkit/documents https://cornell.app.box.com/v/FAIM-tools-resources	Agreement form completed by mentor and mentee that covers values; expectations and learning and development goals; roles and responsibilities; communication channels; progress tracking and feedback; schedule for meetings and agenda; exit strategy Tool designed to align mentor-mentee relationships, which offers a set of guiding principles to initiate discussion at the local and national levels Resource recommended for use at the initiation of a mentoring relationship to allow the mentor and mentee to self-reflect on how each of them understands mentorship and to identify where the mentor’s and mentee’s beliefs converge and diverge
Communications	Mentoring best practices in communication	https://www.medschool.umaryland.edu/media/som/about-us/images/wims/mentor/MentoringBest PracticesCommunication.pdf	Outlines key skills for effective communication in mentoring relationships including awareness, active listening, giving and receiving feedback, and common barriers to effective communication
Organizational tools	Mentor session summary Meeting checklists for mentors and mentees	https://mentoringservices.indiana.edu/resources/summary-form.html https://medicine.iu.edu/faculty/professional-development/mentoring/toolkit/documents	Summary form for mentee to complete following each mentor-mentee meeting
Addressing challenges	Practical resource for how to address challenges	https://ictr.wisc.edu/mentoring/strategies-to-address-mentoring-challenges/	Discusses strategies to effectively address mentoring challenges including providing adequate direction, dealing with conflicting demands and advice
Peer mentoring	Practical Resource	Reference 3	Guidance on Strategies for and Benefits of Peer Mentoring
Evaluations and feedback			
Progress evaluation form	Mentee evaluation (by mentor)	https://hr.nih.gov/sites/default/files/public/documents/working-nih/mentoring/pdf/mentor-mentee-evaluation.pdf	Survey evaluating mentee progress completed by the mentor
Mentor evaluation form	Mentor evaluation tool (by mentee)	https://medschool.duke.edu/sites/default/files/2022-05/Mentee%20 Survey%20Original%20UC%20 Davis_NHLBI.pdf https://uwmadison.qualtrics.com/jfe/form/SV_cZ5jT2DdKYxE66V?Q_JFE=qdg (evaluation of competencies by mentee)	Helps clarify the mentor’s skill and ability to provide academic guidance, communication skills, and role model professional development
Mentor self-evaluation	Mentor self-assessment form (by mentor)	https://medschool.duke.edu/sites/default/files/2021-12/indiana_university_mentor_self_assessment_form.pdf	Self-assessment of mentor’s ability including how well they are you doing in the current mentor–mentee relationship
Mentoring competencies			
Cultural competence	Screening tool of mentor cultural competencies	https://www.evidencebasedmentoring.org/wp-content/uploads/2014/11/Cross-CulturalInventoryRevised-Mentors.pdf	Useful for assessing cultural competency in mentors
Implicit bias assessment	Project implicit bias	https://implicit.harvard.edu/implicit/	Assessments that reveal how these implicit biases can lead people to inadvertently act in ways that may be discriminatory or influenced by stereotypes that people actively reject
Mentorship resources for institutions			
	Developing mentorship training	http://fogartyfellows.org/wp-content/uploads/2015/01/8DZunt.pdf	Discusses steps towards institutionalization of mentorship programs, i.e., provision of mentorship training and mentoring tools
	Clinical mentorship training	https://www.go2itech.org/HTML/CM08/toolkit/overview/index.html	Approaches to clinical mentoring in HIV care and treatment programs and integration across different schools and departments
	Training curriculum for mentors	https://cimerprojectportal.org/#/dashboard	Mentor training materials designed for those wishing to implement professional development workshops for research mentors
	Evaluation of mentorship best practices	https://www.med.unc.edu/aoe/wp-content/uploads/sites/519/2017/12/Evaluation-of-Mentoring-Best-Practices-Tool-Kit-_-with-appended-surveys_2007.pdf	Recommendation of when and how to evaluate the effectiveness of mentorship programs using a range of measurement methods

## MENTORSHIP COMMUNICATION STRATEGIES

Effective communication between mentors and mentees, the cornerstone of successful mentoring relationships, can be enhanced by setting clear expectations. These expectations should be established at the start of a formal mentoring relationship, for example, when creating a mentor–mentee compact. Details may include preferred methods of communication (e.g., E-mail, WhatsApp, or Signal), how often to communicate, a time frame in which to expect a response, and when to follow up.

Effective communication in mentorship relationships requires openness, honesty, active listening, and empathy.^30^ Mentors must make a conscious and deliberate commitment to demonstrate their enthusiasm for the career progression of their mentees through availability to communicate. The issue of mentors’ availability has been previously highlighted and reported to be relatively scarce in comparison with HICs.^1^ From personal experiences of the authors, mentees feel reassured and confident when their mentors, role models, and those who they look up to for guidance demonstrate availability when their support or attention is needed. A simple response to an E-mail from a mentee shows the mentor’s support and commitment to the relationship. Some mentors do not realize or appreciate the negative impact of “ignoring” communications from mentees needing assistance. To better understand these issues, more focused discussion and qualitative research are needed to unpack the critical role of “showing availability” of mentors in mentoring relationships. In circumstances where the mentor is unresponsive to the mentee’s needs, there should be a mechanism to change mentors. Problematic relationships can sometimes be solved by having more than one mentor, either at the same institution or one external (i.e., international) and one local.

Mentors must focus on the quality of communication, particularly when providing feedback. Feedback in mentorship supports learning, improves performance, and can raise a mentee’s awareness of their behavior.^31^ Effective feedback should be constructive and positive to attain the desired effect. The mentor should also be aware of their questioning technique. The funnel questioning technique is a useful framework to help improve a mentor’s ability to probe the issues further to elicit the type of information they may be looking to gain and fuel the discussion.^32^ This framework involves beginning with an “open” question that gives the mentee the opportunity to self-reflect, problem-solve, and provide a comprehensive response. The mentor then follows up with a series of thoughtful and progressively specific questions to glean enough detail for the mentor to have a full understanding, demonstrated by their ability to summarize the conversation.^32^

Given the increase in virtual mentoring during and after the COVID-19 pandemic, it is also important to consider how to maintain effective communication without interacting in person. Virtual mentoring, using platforms such as E-mail, phone calls, or videoconferencing, has provided new opportunities for global health mentoring, including allowing many more mentors, both local and international, to assist mentees with their development. Virtual communication can sometimes feel less personal, but with deliberate structure and consistent engagement, mentors and mentees can collaborate to foster strong relationships and avoid potential challenges to communication and connection. Furthermore, the flexibility of virtual mentoring requires intentional effort to maintain healthy boundaries between personal and academic responsibilities, an important consideration for sustaining effective mentor–mentee relationships.

Despite some challenges, virtual mentoring has many benefits. In the context of COVID-19, it allowed mentoring to continue and led to deepening relationships through shared experiences of the pandemic, including gratitude and resilience. This experience can foster greater enjoyment of the mentoring experience.^33^ For many mentees, virtual mentorship provided an opportunity to continue their research training while fostering interpersonal connection, a sense of purpose through meaningful contributions, and much-needed structure during an otherwise uncertain and disruptive period. Even without in-person interaction, mentors and mentees who worked together to define their goals and expectations could still maintain research productivity. As these endeavors require adaptability and effective communication, institutions would benefit from training the mentors and mentees on best practices for virtual mentoring even in the absence of such a pandemic.^34^

## MENTORING POST FELLOWSHIP

### Transitioning from structured mentorship to professional collegiality.

As research fellowships conclude, it is imperative to transition the mentoring relationship to showcase the mentee’s evolving professional individuality and autonomy. Rather than a sudden termination, ending a formal mentoring relationship can involve mutual reflection, recognition of progress, and a shift toward more peer-like collegiality.^35^ Such a process reinforces the value of the relationship, encourages long-term professional networks, and affirms the mentee’s readiness for independent scholarship and creative works.

Effective strategies for concluding a formal mentoring partnership can include a final structured meeting to review the goals accomplished, provide summative feedback, and discuss future opportunities for collaboration or continued informal mentorship.^35^ Institutions and/or training programs can support this transition by offering guidelines for departure conversations, mentorship feedback, and alumni engagement mechanisms.

A thoughtful conclusion to the mentoring relationship allows the mentor and mentee to mark achievements, realign expectations, and establish a foundation for continued professional association and future collaboration. When managed effectively, the conclusion of a formal mentoring relationship can set the stage for the beginning of long-term professional endeavors.

### Long-term mentoring.

One of the goals of effective mentoring is fostering career progression to independence. The path to achieving this may be daunting without effective communication between the mentee and mentor after a fellowship and related training programs. Often, the mentee may lose career focus or momentum, and their career development could wane without guidance from a mentor. Long-term mentoring is therefore critical for a mentee’s success.

The strategies to maintain career focus needed for growth and career independence of the mentee after completion of the fellowship or training should be openly discussed and agreed upon before the end of the training program. Mentors should consciously check and take responsibility for providing the support needed in this respect.^36^ In many cases, the mentoring relationship will continue, often in a more informal manner, for many years after the completion of a fellowship or training.

One long-term benefit of having the support of an effective mentor is improving the chances of mentees having successful independent research grant support and progression from trainee to instructor, junior faculty, and senior faculty positions.^37^ To achieve this, mentors should create an environment of mutual respect between mentees and mentors, and they should be approachable and willing to provide comprehensive and constructive feedback.^37^ The mentee should complement this with a positive attitude toward self-reflection, receptivity to feedback, and reliability, such as keeping to agreed timelines. Mentoring relationships are bidirectional and require commitment from both parties to maintain a healthy relationship with tangible benefits to all involved.^38^^,^^39^

To sustain the momentum and focus of mentees in their career paths, mentors should make deliberate efforts to sponsor their mentees for career-enhancing activities or programs.^40^ In addition, mentors should be knowledgeable and aware of networking and potential collaborative opportunities for mentees to explore and develop their careers toward research and academic independence.

## STRENGTHENING INSTITUTIONAL MENTORING

Although mentoring relationships occur at the interpersonal level, strengthening mentoring capacity at the institutional level will ensure mentoring efforts are sustainable and wide-reaching. An institutional culture that supports mutual benefits for mentor–mentee relationships is likely to promote long-term successful relationships, especially in LMIC settings.^39^

### Considerations for institutional mentoring in LMICs.

Mentoring programs in high-income settings have been presented as an organized model in comparison with programs in LMICs, which have been described as informal.^1^ Key arguments explaining these differences are founded on the basis that limited resources are allocated for formal mentoring in LMICs, coupled with academic cultures influenced by power dynamics, which are not seen as hospitable to the collaborative ideals of formal mentoring relationships. Although these arguments reflect differing landscapes for fostering structured mentorship programs, they simultaneously present opportunities to develop programs building on the strengths of LMIC researchers to enhance mentoring in these settings.

Monetary and human resources are important factors for the scale and depth of attention given to mentoring in both high- and low-income settings. Mentorship programs cannot be sustained without the availability of mentors. However, university faculty in LMIC settings often hold multiple teaching or research roles to maintain a livable salary, which may not leave protected time for voluntary mentoring of trainees or junior faculty. Although resources to protect faculty time for mentoring may be seen as a luxury and deprioritized, nonmonetary incentives, such as providing awards for exceptional mentorship or including mentorship as a criterion for promotion, may be used.^41^ These strategies have been used successfully in HICs and could likely be adapted for LMIC institutions.^42^ Furthermore, institutions may encourage mentors to publish papers with their trainees and subsequently acknowledge these achievements. Not only does this incentivize mentors to work closely with their mentees to develop advanced writing skills, but it benefits both the trainee and mentor by strengthening their publication records, an important factor in being awarded research grants and academic promotions.

Compounding the above point is the reality that many senior academics in LMICs have not been mentored in formal or structured mentorship programs. Rather, they were likely successful because of informal mentoring relationships they maintained. Institutions, therefore, may be less likely to invest in formal mentoring programs, particularly when resources are limited. However, the existence of informal mentoring relationships shows interest and a clear need for mentoring.

Formal mentorship programs also contend with prominent cultural factors that can create barriers to mentorship. There is a hierarchy in many low-income settings that promotes paternalism, requires acceptance of a senior’s ideas, and discourages critical thinking or challenging the mentor.^1^ In settings where the use of formal titles is expected, collaborative and collegial relationships between mentors and mentees may be challenging due to the inherent power dynamics. This practice also hinders respectful disagreement and questioning of senior faculty members, which are important for mentees to grow.^43^ This culture of expected “respect” or “reverence” could be counterproductive to achieving mentoring goals, including progress toward career independence. Although this challenge is prominent in LMIC settings, the barrier persists in high-income settings as well. In the United States, postdoctoral researchers have cited academic politics and scientific hierarchy as a structural barrier to successful mentoring.^44^ In both high- and low-income settings, successful mentoring relationships promote openness to mentoring up, where the mentee co-directs the relationship, including setting goals, planning agendas, and requesting support and feedback as needed.^45^ This collaborative approach provides the basis for a satisfying and productive mentoring relationship and facilitates the mentor’s participation in the relationship.^45^ Taking into account how busy most mentors are, proactive mentees who schedule the next meeting at the end of a mentoring encounter are more likely to receive more mentoring. Encouraging mentoring up, even in settings with strict hierarchy, would enable the mentee to check the mentor’s priorities and expectations and guide the relationship to fit their needs, even in the context of power imbalances.

With the above analysis in mind, significant opportunities exist to strengthen mentoring in low-income settings by building on existing strengths and addressing common challenges. These opportunities should be centered on encouraging mentorship of trainees and junior researchers, particularly to ensure they are building positive mentoring relationships.

### Developing a strategic framework to guide institutional mentoring initiatives in LMICs.

Some universities have institutionalized mentorship, and there is much we can learn from key examples (Box 2). The programs in Ethiopia and Kenya illustrate the importance of long-term planning for sustainability, as well as programmatic and individual factors contributing to success. Building on these learnings and suggestions from Lescano et al.,^1^ we propose the following elements for successful institutional efforts:

1. **Responsibility and ownership of institutional mentoring will improve the impact of mentorship programs.** Institutions could establish an office and a small team that has mentoring as a priority to oversee and manage mentoring activities. Ideally this should come with some level of financial support, but making this happen requires internal advocacy with the institution’s administration. This administrative structure formalizes mentoring and ensures that staff are responsible for implementing these activities, including producing written expectations for mentors and mentees and institutional guidance for mentoring. One relevant HIC example is the mentoring guidelines for postdoctoral fellows at the Harvard T.H. Chan School of Public Health Office of Faculty Affairs, which formalizes the processes and expectations.^46^
2. **Establishing clear program activities, goals, and requirements for participation promotes successful mentorship.** A process-based approach in mentorship enables the mentor to communicate, align expectations, assess understanding, address equity and inclusion, foster independence, and promote professional development of the mentee.^47^ To build institutional mentoring capacity, one needs to understand who the participants are, where they are, their developmental needs, and what motivates them. Institutional mentoring programs that lack the desired success, including in higher education, often lack clear goals or clarified benefits at individual and organizational levels.^48^ A well-planned mentoring program will encourage participants to engage with others on a personal level and build strong, trusting relationships that motivate and guide toward future goals. In this regard, a multidimensional framework to support these programs’ development, implementation, and assessment must be established. In addition to communicating clear values, guidelines for mentoring activities should be conveyed clearly, for example on the frequency of mentor–mentee meetings or expected deliverables such as coauthored publications. Additionally, clarifying requirements for participation is important for success. Academic institutions may require mentors to be faculty members or professionals who are willing to commit to the mentor program.^49^
3. **Matching mentors and mentees should be a core component for institutional mentoring programs.** A system can be designed to match mentors with mentees based on their mutual areas of academic or professional interest, geographic proximity, and schedules.^50^ An approach reported by Oppong et al. is pre-mentoring, a process in which mentors and mentees “can explore and match interests before initiating a mentoring relationship.”^51^ Brizuela et al. noted that matching based on specific criteria resulted in a high level of satisfaction in the mentoring relationship, and they received feedback that mentees would prefer to have information about the potential mentors before matching.^52^ Although programs have many approaches to matching mentors and mentees, power and gender dynamics should be taken into account.
  Institutions may consider assigning multi-mentor teams to provide support in different ways. Models may include assigning primary and secondary mentors for incoming tenure-track faculty and mentoring committees for postdoctoral fellows. In HICs, mentoring is typically viewed as separate from academic supervision, although this is often not differentiated in LMIC settings. Building mentoring into working relationships can be beneficial; however, it is also important to include mentors who are not supervisors involved in the faculty member’s work.^44^
4. **Mentor and mentee training is a key component to improve the efficacy of institutional mentoring.** Mentors generally approach mentoring without any prior training, leading them to mentor students and trainees based on what they observed from other mentors and by trial and error. Formal global health mentorship programs, such as the HRP Alliance mentorship program, have implemented training for mentors and mentees at the beginning of the relationship and provided opportunities for ongoing training and support throughout.^52^ Mentor training was conducted over 1 day and focused on building rapport with mentees, communication, identifying barriers to career progression, and clarifying the mentor-mentee relationship. Programs without formalized mentor training can enhance mentorship experiences by holding periodic intensive mentoring workshops, which may be a feasible option for institutions with limited resources. Overall, limited formal training opportunities are available for mentors, particularly in LMICs. Addressing this challenge can facilitate optimal training experiences for mentees.
5. **Resources, support, and recognition for mentors should be included to encourage active engagement and acknowledge their time and effort.** When possible, monetary compensation for mentors should be provided to ensure protected time for mentorship activities. Including mentorship as a requirement for promotion, encouraging joint publications, and giving awards for mentorship can also be compelling incentives.
6. **Monitor and evaluate the mentoring program’s results.** To ensure that a mentoring program meets its objectives, it is important to maintain open communication with all participants and actively solicit feedback on the results. Evaluations based on this feedback can assist current participants in achieving their goals for the program and improve the mentor program for future mentors and mentees. Actively involving mentors in this process can also serve as a strategy to encourage continued mentor engagement. Publishing results of program evaluations will provide a strong evidence base to contribute to the continued growth of institutional mentoring.

**Box 2:** Mentorship-driven postgraduate training: Case studies from Ethiopia and Kenya

We present two illustrative case studies of structured postgraduate mentorship programs designed to strengthen research capacity and training in public health and related fields in sub-Saharan Africa. These examples—from Ethiopia and Kenya—highlight different models for implementing academic mentorship at scale, ranging from university-based consortiums to national networks. While tailored to specific institutional and national contexts, both programs share core principles such as co-supervision, faculty development, structured feedback, and academic enculturation. The aim of this summary is not to present a uniform model, but instead to showcase effective, mentorship-driven strategies that have improved postgraduate training outcomes, supported academic retention, and fostered research productivity in resource-constrained settings. Ethiopia – Postgraduate Mentorship Program Overview Ethiopia’s mentorship initiative, led by the Addis Continental Institute of Public Health (ACIPH), involves a network of 10 public universities and has been ongoing since 2008. Though implemented with minimal external funding, the program has built a strong cadre of local academic leaders and contributed to the expansion of graduate-level public health training programs nationwide. Elements of the program entailed: Phased National Roll-Out: The program began with formal agreements among partner universities and prioritized investment in mentoring junior faculty during and after their PhD training. Mentor Pairing across Ranks: Junior faculty were first mentored by ACIPH senior staff. Once trained, they co-supervised PhD students and independently mentored MPH students, creating a pipeline of in-country mentors. Peer Support and Collaborative Supervision: Cross-institutional collaboration enabled shared teaching and student supervision, promoting inter-university learning and reducing isolation. Mentorship in Grant Writing and Research: Faculty were coached in grant development, and many went on to secure competitive research funding—building institutional credibility and resources. Access to Global Networks: Through ACIPH’s partnerships, mentees accessed international mentorship opportunities and short-term trainings to build advanced technical capacity. Kenya – Postgraduate Mentorship Program Overview Kenya’s postgraduate mentorship program, supported by the Fogarty International Center (FIC), is implemented across several universities including Kenyatta, Maseno, MMUST, JOOUST, and Kirinyaga. Its objective is to build postgraduate research capacity through structured mentorship, with a strong focus on timely program completion, academic writing, and integration into scholarly communities. The mentorship structure includes: Early Research Orientation: Mentees are introduced to scientific literature and academic discussions early to help develop researchable questions, clear objectives, and conceptual thinking. Multidisciplinary, Hands-On Support: Mentors offer practical guidance in research design, data collection, and analysis. Weekly meetings with multidisciplinary experts provide consistent feedback and track progress. International Exposure: When local technical capacity is limited, mentors facilitate access to international laboratory placements and collaborations, offering mentees advanced methodological exposure. Academic Networking and Enculturation: Mentors encourage participation in conferences, workshops, and seminars. These experiences promote academic identity and long-term engagement with the scholarly community. Joint Feedback and Co-Supervision: Structured co-supervision involves mentors from both the host institution and the program, ensuring consistent, coordinated academic guidance across disciplines. Academic Writing and Publication: Mentors support mentees in reading, analyzing, and eventually publishing scientific work, often starting through co-authored papers. Lessons Learned from Ethiopia and Kenya: Regular mentor-mentee engagement, including timely, constructive feedback—supports research quality, independent thinking, and completion rates. Co-supervision and structured peer networks improve collaboration, consistency, and overall research experience. Long-term mentorship investment builds institutional sustainability and reduces dependence on external expertise. Mentorship promotes academic integration by helping students and faculty form scholarly identities and develop professional networks. Training in research methods, writing, and grant development prepares mentees to become competitive and independent researchers. Effective mentorship can be achieved even with limited funding when driven by local leadership, institutional collaboration, and shared commitment. The mentorship programs in Ethiopia and Kenya demonstrate how structured, locally driven models can effectively strengthen postgraduate training and research capacity in Africa. Through strong mentor-mentee relationships, co-supervision, research skill-building, and academic enculturation, both programs have produced high-quality graduates, enhanced institutional capacity, and retained talent in-country. These models offer practical approaches for addressing similar challenges in other African contexts. Several factors contributed to success across both programs. Strong institutional leadership and coordinated networks created well-defined systems for mentor–mentee pairing and phased training. Committed mentors provided flexible, personalized academic support, while early communication agreements fostered accountability. Peer learning was supported through joint supervision and inter-university collaboration. Faculty retention and locally anchored mentorship strengthened continuity. Emphasis on real-world research, competitive grant writing, and publishing aligned mentorship with institutional priorities. Finally, the strategic use of global mentor networks expanded training without heavy reliance on external funding—demonstrating how mentorship can thrive in low-resource settings and build the next generation of African scholars.

## CONCLUSION

Mentoring in global health is a foundational strategy for building individual research capacity and strengthening academic communities. At every stage of the mentorship journey, from developing core skills and effective communication to guiding mentees through publication, career transitions, and institutional integration, structured and intentional support fosters more meaningful, productive, and lasting relationships. However, challenges such as unequal access to resources, contextual differences across global health settings, and organizational barriers must be actively addressed by both individuals and institutions.

Looking ahead, sustained investment in mentoring is essential. Priorities should include capacity-building for mentors and mentees, the development of adaptable yet structured mentorship models, integration of mentoring into institutional frameworks, and the creation of systems for recognition, evaluation, and continuous support. Strengthening mentorship requires shared responsibility and collaboration, with individual mentors and institutions working together to learn from experience, share effective practices, and jointly advance mentorship in global health research.
